# Supplementary material for: The NanoBridge system for targeted post-translational modification of challenging proteins
Source: bioRxiv. 2026 Jun 10:2026.06.08.730974. Preprint. [Version 1] doi: 10.64898/2026.06.08.730974 (PMC13278106; doi:10.64898/2026.06.08.730974)
Supplement: Supplementary file 1 [file NIHPP2026.06.08.730974v1-supplement-1.pdf]

bioRxiv preprint doi: https://doi.org/10.64898/2026.06.08.730974; this version posted June 10, 2026. The copyright holder for this preprint (which was not certified by peer review) is the author/funder, who has granted bioRxiv a license to display the preprint in perpetuity. It is made available under a CC-BY 4.0 International license.

Supporting information for

The NanoBridge system for targeted post-translational modification of challenging proteins

Fangfang Shen[1], Olga D. Merino-Chavez[2], Sheng-Yao Dai[3], Sebastian Alfonso[1], Laura M. K. Dassama[1,4*]

[1]Department of Chemistry and Sarafan ChEM-H Institute, Stanford University, Stanford, California 94305, United States.

[2]Department of Chemical and Systems Biology, Stanford University School of Medicine, Stanford, California 94305, United States.

[3]Department of Radiology, Molecular Imaging Program at Stanford, Stanford University School of Medicine, Stanford, California 94305, United States.

[4]Department of Microbiology & Immunology, Stanford School of Medicine, Stanford, California 94305, United States.

[*] Correspondence to: dassama@stanford.edu

bioRxiv preprint doi: https://doi.org/10.64898/2026.06.08.730974; this version posted June 10, 2026. The copyright holder for this preprint (which was not certified by peer review) is the author/funder, who has granted bioRxiv a license to display the preprint in perpetuity. It is made available under a CC-BY 4.0 International license.

Table of Contents

1. Materials and Methods *3*
Cell culture. 3
Plasmid construction 4
Transformation 4
Lentivirus productions and transduction 5
Immunoblotting. 5
RT-qPCR 6
Confocal imaging 7
Flow cytometry to analyze KRAS degradation. 7
Immunoprecipitation and proteomics analysis 8
Statistics 9
2. Protein sequences of NanoBridge constructs. *10*
3. Supplementary figures. *13*
Figure S1. 15
Figure S2. .... **Error! Bookmark not defined.**
Figure S3. .... **Error! Bookmark not defined.**
Figure S4. .... **Error! Bookmark not defined.**
Figure S5. .... **Error! Bookmark not defined.**
4. Supplementary tables *17*
Table S1. Primers used for plasmid construction 17
Table S2. Primes used for RT-qPCR 18
Table S3. C_q values of hemoglobin transcripts in $CD34^+$ cells. 19
5. References. *20*

bioRxiv preprint doi: https://doi.org/10.64898/2026.06.08.730974; this version posted June 10, 2026. The copyright holder for this preprint (which was not certified by peer review) is the author/funder, who has granted bioRxiv a license to display the preprint in perpetuity. It is made available under a CC-BY 4.0 International license.

1. Materials and Methods

Cell culture

Cells were cultured in a humidified atmosphere of 5% CO_2 and 95% air at 37°C. Mycoplasma tests were carried out routinely to detect contamination. For cell counting and viability measurement, the cell suspension was mixed 1:1 with 0.4% trypan blue dye (Thermo Scientific, #15250061) and measured with Countess II FL Automated Cell Counter (Thermo Scientific).

HUDEP-2 cells (RCB4557) were obtained from Riken Bio Resource Research Center (Japan). Cells were cultured according to the reported method.[1] In brief, cells were maintained in expansion medium, which contains StemSpan serum-free expansion medium (SFEM, StemCell Technologies, #09650), 2% Penicillin-Streptomycin solution (10,000 U/mL stock), 50 ng/mL recombinant human stem cell factor (SCF, StemCell Technologies, #78062.2), 3 IU/mL Epoetin alfa (EPO, Sigma, #H5166), 0.4 μg/mL dexamethasone (Sigma, #D4902), and 1 μg/mL doxycycline (Thermo Scientific, # BP26531).

Peripheral blood-derived $CD34^+$ cells from multiple donors were obtained from the NIDDK-Center of Excellence in Hematology at the Fred Hutchinson Cancer Research Center. The cells were cultured according to the reported method.[2] In brief, cells were cultured in erythroid differentiation medium (EDM), which contains IMDM medium (Gibco, #12440053) supplemented with stabilized glutamine, 330 ug/mL holo-human transferrin (Sigma, #T0665), 10 ug/mL recombinant human insulin (Sigma, #I9278), 2 IU/mL heparin (StemCell Technologies, #07980), and 5% inactivated human plasma type AB (Sigma, #H5667). The expansion procedure comprised 3 steps. In the first step (Day 0 to Day 7), 10^4/mL $CD34^+$ cells were cultured in EDM in the presence of 10^{-6} M hydrocortisone (StemCell Technologies, #07925), 100 ng/mL SCF, 5 ng/mL IL-3 (StemCell Technologies, #78146), and 3 IU/mL EPO. On Day 4, 1 volume of cell culture was diluted in 4 volumes of fresh medium containing SCF, IL-3, EPO, and hydrocortisone. In the second step (Day 7 to Day 11), the cells were resuspended at 10^5/mL in EDM supplemented with SCF and EPO. In the third step (Day 11 to Day 13), the cells were cultured in EDM supplemented with EPO alone. Cell counts were adjusted to a range of 7.5×10^5 - 1×10^6 on Day 11.

Human HEK293T cells (female) were purchased from ATCC. The cells were cultured in DMEM supplemented with 10% FBS, 1% Penicillin-Streptomycin solution (10,000 U/mL stock).

bioRxiv preprint doi: https://doi.org/10.64898/2026.06.08.730974; this version posted June 10, 2026. The copyright holder for this preprint (which was not certified by peer review) is the author/funder, who has granted bioRxiv a license to display the preprint in perpetuity. It is made available under a CC-BY 4.0 International license.

MIA PaCa-2 cells (CRL-1420) were purchased from ATCC. The cells were cultured in DMEM supplemented with 10% FBS, 2.5% horse serum, and 1% Penicillin-Streptomycin solution (10,000 U/mL stock).

HCT116 cells (CCL-247) were purchased from ATCC. The cells were cultured in McCoy's 5a Medium Modified (Thermo Scientific, #16600082), supplemented with 10% FBS, and 1% Penicillin-Streptomycin solution (10,000 U/mL stock).

A549 cells was a gift from Dr. Jiang Hong Rao lab at Stanford University School of Medicine. The cells were cultured in DMEM, high glucose (Thermo Fisher Scientific, #11965), supplemented with 10% FBS and 1% Penicillin-Streptomycin solution (10,000 U/mL stock).

H1299 cells was a gift from Dr. Maximillian Diehn lab at Stanford University School of Medicine. The cells were cultured in RPMI 1640 Medium (Thermo Scientific, #11875093), supplemented with 10% FBS and 1% Penicillin-Streptomycin solution (10,000 U/mL stock).

Plasmid construction

All gene blocks were obtained from GENEWIZ while primers were obtained from Integrated DNA Technologies. KOD hot start polymerase (Sigma, #71086-3) was used for PCR reaction, the primers used are listed in Table S1. The PCR products was digested with DPNI (NEB, #R0176L) and purified with PCR purification kit (Invitrogen, #K310001). NEBuilder HiFi DNA Assembly (NEB, #E2621X) was used for plasmid fusion.

Transformation

2 µL of plasmid (concentration ≥20 ng/µL) was added to a thawed tube of Stellar competent cells (Takara, #636763) and incubated on ice for 15 min. The cells were heat shocked at 42 °C for 45 s and placed on ice for 2 min. 900 µL of LB media were added and the cells cultured for 1 h at 37 °C while shaking at 200 rpm. Cells were centrifuged for 3 min at 2500 × g and 900 µL LB media was removed. The recovered cells were resuspended in 100 µL remaining media and added to ampicillin-(100 mg/mL) or kanamycin-containing (50 mg/mL) agar plates and incubated at 37 °C overnight.

bioRxiv preprint doi: https://doi.org/10.64898/2026.06.08.730974; this version posted June 10, 2026. The copyright holder for this preprint (which was not certified by peer review) is the author/funder, who has granted bioRxiv a license to display the preprint in perpetuity. It is made available under a CC-BY 4.0 International license.

Lentivirus productions and transduction

To generate NanoBridge expressing cell lines, the corresponding NanoBridge sequence were subcloned into the lentiviral vector pLVX-EF1a-IRES-Puro (Addgene, #85132). Lentiviruses were packaged in HEK293T cells as described previously.[3] In brief, 1.25 µg plasmid with gene of interest, 0.8 µg psPAX2 (Addgene, #12260) and 0.45 µg pMD2.G (Addgene, #12259) were added into 50 µl of Opti-MEM medium (Thermo Scientific, #31985062). At the same time, 7.25 µg PEI (Polysciences Inc) was added into another 50 µl of Opti-MEM medium. After incubation for 3 min, the plasmid containing medium was added into PEI medium, mixed well and incubated for 15 to 25 min. The mixture was added dropwise into a HEK293T cells cultured in 6-well plate. 12 h later after transfection, medium was replaced with fresh complete DMEM medium. After another 48h, the supernatant was collected and filtered through 0.45 µM filter to give the lentivirus. The lentivirus can be used fresh or save in -80°C for later use.

For transduction, 1 mL of lentivirus containing medium was added to cells cultured in 1 mL of complete medium, incubate for 24 - 48 h. After that medium was changed to fresh complete medium, and cells were cultured for another 2 days. After that, puromycin at the concentration of 1 or 2 µM was used to select the stable expressing cells.

For the transduction of $CD34^+$ cells, cells were cultured as the method described above. On Day 3, $CD34^+$ cells were incubated with lentivirus containing medium at the ratio of 1:1 and incubated for 48h. On Day 4, the medium was changed to complete $CD34^+$ differentiation medium. On Day 5, puromycin at the concentration of 1µg/mL was added to select for the NanoBridge stable expressing cells. The treatment of puromycin was continued until the end of this experiment. From Day 8 to Day 13, cells were treated with dTAG47 at the concentration of 500 nM. Cells from Day 8 to Day 13 were harvested and analyzed.

Immunoblotting

For degradation studies, cells were washed with cold DPBS buffer and lysed using RIPA buffer (VWR, #97063-270) containing 0.1 U Benzonase nuclease (Santa Cruz biotechnology, #sc-391121B) and EDTA-free protease inhibitor cocktail (Bimake, #B14002). For acetylation studies, cells were washed with cold DPBS buffer and lysed using RIPA buffer containing 0.1 U Benzonase

bioRxiv preprint doi: https://doi.org/10.64898/2026.06.08.730974; this version posted June 10, 2026. The copyright holder for this preprint (which was not certified by peer review) is the author/funder, who has granted bioRxiv a license to display the preprint in perpetuity. It is made available under a CC-BY 4.0 International license.

nuclease, EDTA-free protease inhibitor cocktail, 5 mM sodium butyric acid, and 10 mM nicotinamide. Cellular debris was cleared by centrifugation at 16,000 g for 20 min at 4°C. Protein extracts were quantified by Pierce BCA Protein Assay Kit (Thermo Scientific, #PI23235) with a Nanodrop One^{C} (Thermo Scientific) according to the manufacturer's instruction. Protein lysates (~ 30 µg/lane) were resolved by SDS-PAGE and transferred onto poly vinylidene difluoride (PVDF) membranes. Membranes were blocked with 5% non-fat milk in 0.1% Tween 20/PBS for 1 h. The blots were then probed with the relevant primary antibodies in blocking solution at 4°C overnight with gentle agitation. Membranes were washed 5 min with 0.1% Tween 20 in PBS three times and were incubated with secondary antibodies for 1 h at room temperature. For HRP-conjugated secondary antibody, antigens were detected by addition of Clarity™ Western ECL Substrate (Bio-Rad, #1705060). The membranes were visualized by chemiluminescence or fluorescence on a Bio-Rad ChemiDoc MP Imaging System. Primary antibodies used: BCL11A (Cell Signaling, #75432), StrepMAB-Classic HRP (IBA, #2-1509-001), GAPDH (Santa Cruz Biotechnology, #sc-365062), Lamin b1 (Proteintech, #66095-1), β-tubulin (Proteintech, #66240-1), β-hemoglobin (Sigma Aldrich, #WH0003043M1), g-hemoglobin (Proteintech, #25728-1-AP). Flag tag (Cell signaling, #14793), KRAS (Proteintech, #12063-1-AP), p53 (Santa Cruz Biotechnology, #sc-126), p53-K382Ac (Cell Signaling, #2525), p53-K305Ac (Abcam, #Ab109396), p53-K320Ac (Sigma, #06-1283), p53-K370Ac (Abcam, #Ab183544), p53-K373Ac (Abcam, #Ab62376), p53-K386Ac (Abcam, #Ab52172). Secondary antibodies used: Anti-Mouse-HRP (Abcam, #ab6728), Anti-Rabbit-HRP (Promega, #W4018), IRDye 680RD Goat anti-Mouse IgG Secondary Antibody (LI-COR, #926-68070), IRDye 880RD Goat anti-Rabbit IgG Secondary Antibody (LI-COR, #926-32213).

RT-qPCR

Quantitative real-time PCR was performed to quantify RNA abundance. For each sample, total RNA was isolated by using the RNeasy Plus Mini Kit (QIAGEN Cat# 74134), followed by cDNA synthesis using the iScript cDNA Synthesis Kit (BioRad, Cat# 1708890). qRT-PCR primers (Table S2) were ordered from Integrated DNA Technologies. Quantitative real-time PCR was performed using the PrimePCR assay with the iTaq universal SYBR Green supermix (BioRad, #1725120) and run on a Biorad CFX384 real-time system (C1000 Touch Thermal Cycler) according to the

bioRxiv preprint doi: https://doi.org/10.64898/2026.06.08.730974; this version posted June 10, 2026. The copyright holder for this preprint (which was not certified by peer review) is the author/funder, who has granted bioRxiv a license to display the preprint in perpetuity. It is made available under a CC-BY 4.0 International license.

manufacturer's instructions. C_q values were used to quantify RNA abundance. The relative abundance of the hemoglobin was normalized to a GAPDH internal control by using this equation:

$$\Delta Cq = Cq\ (\text{gene of interest}) - Cq\ (\text{GAPDH}),$$
$$R = 2^{-\Delta Cq}$$

Representative data and calculations are shown in Table S3.

Confocal imaging

200 µL of HEK293T cells expressing K27-FKBP12^{F36V} were seeded into 8-well chamber slides (Ibidi, #80807) at a density of 2×10^5 cells/mL. Cells were cultured for 24 h at 37 °C. After that, cells were treated with DMSO, dTAG^{V}-1 500 nM or dTAG^{V}-1 (500 nM) + MG132 (500 nM) for another 24 h. After treatment, cells were washed with cold PBS buffer once and fixed by addition of 4% paraformaldehyde for 20 min at 4 ºC. Then, cells were permeabilized by adding 0.1% Triton-X100 in PBS for 5 min. After that, cells were blocked with 5% BSA in 0.1% Tween 20/PBS for 1h and incubated with anti-FLAG and anti-KRAS antibodies in 1% BSA in 0.1% Tween 20/PBS overnight. Following that, cells were washed with 0.1% Tween 20/PBS for 3 times, and incubated with 1 µg/mL DAPI, secondary antibodies Goat anti-rabbit IgG (H+L) -488 (Invitrogen, #A32731) and Goat anti-Mouse-IgG (H+L) -Alexa Fluor 647 (Invitrogen, #A21235) for 1h at room temperature. Then, cells were washed 3 times with PBST and imaged with a Zeiss LSM980 Confocal Microscope with 60 × oil immersion objective at excitations of 653, 493 and 353 nm.

Flow cytometry to analyze KRAS degradation

2mL of HEK293T cells expressing K27-FKBP12^{F36V} were seeded into 6-well plate at the density of 2×10^5 cells/mL. Cells were cultured for 24 h at 37 °C. After that, cells were treated with dTAG^{V}-1 at the indicated concentrations for 24 h. After treatment, cells were harvested and washed twice with cold PBS buffer and centrifuged at 350-500 × g for 5 min. Then, cells were fixed with 4% paraformaldehyde for 20 min at 4 ºC and permeabilized with 0.2 % Triton-X100 in PBS for 5 min. Following that, 100 µL of 0.1% BSA in 0.1% Tween 20/PBS buffer was added for blocking, and cells were incubated with anti-KRAS or anti-FLAG for 2 h, washed 3 times with 0.1% Tween 20 in PBS buffer and incubated with Goat anti-rabbit -Alexa Fluor 647 (Invitrogen, #A21244) for 1 h at room temperature. After washing 3 times with 0.1% Tween 20/PBS buffer, cells were

bioRxiv preprint doi: https://doi.org/10.64898/2026.06.08.730974; this version posted June 10, 2026. The copyright holder for this preprint (which was not certified by peer review) is the author/funder, who has granted bioRxiv a license to display the preprint in perpetuity. It is made available under a CC-BY 4.0 International license.

analyzed on a BD Accuri™ C6 Plus cell sorter and analyzer. The data was analyzed by FlowJo software.

Immunoprecipitation and proteomics analysis

H1299 Cells were cultured in 10 cm dishes in indicated media. DMSO or AceTAG-1 (500 nM) were added once the cells grew to 70-80% confluency and incubated for 16 h. The cells were harvested and washed with cold PBS twice. The pelleted cells were lysed in 1 mL RIPA lysis buffer, containing 5 mM sodium butyrate, 20 mM nicotinamide, and 1× protease inhibitor (Selleckchem, #B14001), and 0.1 U Benzonase nuclease for 25 min at 4°C. Following that, the cell lysate was centrifugation at 16,000 × *g* for 20 min at 4°C. The protein concentration was normalized after assessing the total protein concentration using the BCA Protein Assay kit (Thermo Scientific, #23227), equivalent amount of protein was subjected to enrichment by p53 antibody. Enrichment was carried out at 4°C for 8h while rotating. During incubation, the magnetic protein A/G beads (Thermo Scientific, #88802) were prewashed with lysis buffer for 3 times. After the primary antibody incubation, magnetic protein A/G beads were added into cell lysate and incubate for another 4h. The beads were enriched by magnetic rack and washed with lysis buffer and cold PBS.

Protein samples on magnetic beads were washed four times with 200 µL of 50 mM Triethyl ammonium bicarbonate (TEAB) with a twenty-minute shake time at 4℃ in between each wash. Roughly 2.5 µg of trypsin was added to the bead and TEAB mixture, and the samples were digested over night at 800 rpm shake speed, at 37°C, followed by a boost addition of trypsin using same wt/wt ratios for overnight digestion at 37°C. To stop the digestion, the reaction mixture was acidified with 1% trifluoroacetic acid (TFA). The supernatant containing the tryptic peptides, was dried in a vacuum centrifuge and re-constituted in water with 2% acetonitrile (ACN).

500 ng total peptide was loaded onto a disposable Evotip C18 trap column (Evosep Biosytems, Denmark) and subjected to nanoLC on a Evosep One instrument (Evosep Biosystems). Tips were eluted directly onto a PepSep analytical column, dimensions: 150 µm x 25 cm C18 column (PepSep, Denmark) with 1.5 µm particle size (100 Å pores) (Bruker Daltronics). Mobile phases A and B were water with 0.1% formic acid (v/v) and 80/20/0.1% ACN/water/formic acid (v/v/v), respectively. The standard pre-set method of 60 samples-per-day (21minute run) was used. The mass spectrometry was done on a hybrid trapped ion mobility spectrometry-quadrupole time of

bioRxiv preprint doi: https://doi.org/10.64898/2026.06.08.730974; this version posted June 10, 2026. The copyright holder for this preprint (which was not certified by peer review) is the author/funder, who has granted bioRxiv a license to display the preprint in perpetuity. It is made available under a CC-BY 4.0 International license.

flight mass spectrometer (timsTOF HT, Bruker Daltonics, Bremen, Germany), operated in PASEF mode. The acquisition scheme used for DIA consisted of 36 precursor windows at width of 25m/z, over the mass range 300-1200 Dalton. The TIMS scans layer the doubly and triply charged peptides.

LCMS files were processed with Spectronaut v20 (Biognosys, Zurich, Switzerland) using DirectDIA analysis mode. Mass tolerance/accuracy for precursor and fragment identification was set to default settings. The reviewed FASTA for *Homo sapiens*, UP00005640, downloaded from Uniprot (on 28/02/2025) and a database of 380 common laboratory contaminant proteins (https://www.thegpm.org/crap/) were used. Methionine oxidation and acetylation of protein N termini were set as variable modifications. A decoy false discovery rate (FDR) at less than 1% for peptide spectrum matches and protein group identifications was used for spectra filtering (Spectronaut default). Decoy database hits, proteins identified as potential contaminants, and proteins identified exclusively by one site modification were excluded from further analysis.

For data analysis, the acetylated peptide was normalized to total peptide abundance, and the fold change of normalized acetylated peptide in AceTAG-1 treated group *vs* DMSO treated group was calculated and presented in figure. Note that peptides may not have been detected in every replicate but were required to be detected and quantified in at least two biological replicates to be included in this analysis. And for a same site, different peptide isoforms may be detected and are calculated separately.

Statistics

All data presented were mean ± SD. Statistical analysis was performed by using Prism 10 and p values obtained from one-way ANOVA, two-way ANOVA or unpaired Student's t-test where necessary; ns, not significant; *p < 0.05; **p < 0.01; ***p < 0.001; ****p < 0.0001.

bioRxiv preprint doi: https://doi.org/10.64898/2026.06.08.730974; this version posted June 10, 2026. The copyright holder for this preprint (which was not certified by peer review) is the author/funder, who has granted bioRxiv a license to display the preprint in perpetuity. It is made available under a CC-BY 4.0 International license.

2. Protein sequences of NanoBridge constructs

Nb6101.16-FKBP12^{F36V}-NLS-Strep tag

MRVQLVESGGGLVQAGGSLRLSCAADGFDFKSYAMGWYRQAPGREDELVAAITASGDY
TYYADSVKGRFTISRDNAKNTVYLQMNSLKPDDTAVYYCAALSYVAEGYWGQGTQVT
VSSGSGSGSMGVQVETISPGDGRTFPKRGQTCVVHYTGMLEDGKKVDSSRDRNKPFKF
MLGKQEVIRGWEEGVAQMSVGQRAKLTISPDYAYGATGHPGIIPPHATLVFDVELLKLEG
SKRPAATKKAGQAKKKKGGGSWSHPQFEKGPV

2D9-FKBP12^{F36V}-NLS-Strep tag

MQVQLVESGGGLVQAGGSLRLSCAASGSIFVNNAMGWYRQAPGKERELVAAISASGGS
TYYADSVKGRFTISRDNAKNTVYLQMNSLKPEDTAVYYCAADQDGYPYEYWGQGTQV
TVSSGSGSGSMGVQVETISPGDGRTFPKRGQTCVVHYTGMLEDGKKVDSSRDRNKPFK
FMLGKQEVIRGWEEGVAQMSVGQRAKLTISPDYAYGATGHPGIIPPHATLVFDVELLKLE
GSKRPAATKKAGQAKKKKGGGSWSHPQFEKGPV

45S-FKBP12^{F36V}-NLS-Strep tag

MRVQLVESGGGLVQAGGSLRLSCAASGFIFDSYAMGWYRQAPGKESELVAAITSSGSST
YYADSVKGRFTISRDNAKNTVYLQMNSLKPEDTAVYYCAALDYVIDGYWGQGTQVTV
SSGSGSGSMGVQVETISPGDGRTFPKRGQTCVVHYTGMLEDGKKVDSSRDRNKPFKFM
LGKQEVIRGWEEGVAQMSVGQRAKLTISPDYAYGATGHPGIIPPHATLVFDVELLKLEGS
KRPAATKKAGQAKKKKGGGSWSHPQFEKGPV

K19-FKBP12^{F36V}-3×FLAG tag

MDLGKKLLEAARAGQDDEVRILMANGADVNASDRWGWTPLHLAAWWGHLEIVEVLL
KRGADVSAADLHGQSPLHLAAMVGHLEIVEVLLKYGADVNAKDTMGATPLHLAARSG
HLEIVEELLKNGADMNAQDKFGKTTFDISTDNGNEDLAEILQKLGSGSGSMGVQVETISP
GDGRTFPKRGQTCVVHYTGMLEDGKKVDSSRDRNKPFKFMLGKQEVIRGWEEGVAQM
SVGQRAKLTISPDYAYGATGHPGIIPPHATLVFDVELLKLEASDYKDDDDKGDYKDDDD
KGDYKDDDDK

bioRxiv preprint doi: https://doi.org/10.64898/2026.06.08.730974; this version posted June 10, 2026. The copyright holder for this preprint (which was not certified by peer review) is the author/funder, who has granted bioRxiv a license to display the preprint in perpetuity. It is made available under a CC-BY 4.0 International license.

FKBP12^{F36V}-K19-3×FLAG tag

MGVQVETISPGDGRTFPKRGQTCVVHYTGMLEDGKKVDSSRDRNKPFKFMLGKQEVIR
GWEEGVAQMSVGQRAKLTISPDYAYGATGHPGIIPPHATLVFDVELLKLEGSGSGSGSDL
GKKLLEAARAGQDDEVRILMANGADVNASDRWGWTPLHLAAWWGHLEIVEVLLKRG
ADVSAADLHGQSPLHLAAMVGHLEIVEVLLKYGADVNAKDTMGATPLHLAARSGHLEI
VEELLKNGADMNAQDKFGKTTFDISTDNGNEDLAEILQKLASDYKDDDDKGDYKDDD
DKGDYKDDDDK

K27-FKBP12^{F36V}-3×FLAG tag

MDLGKKLLEAARAGQDDEVRILMANGADVNAHDTFGFTPLHLAALYGHLEIVEVLLKN
GADVNADDSYGRTPLHLAAMRGHLEIVEVLLKYGADVNAADEEGRTPLHLAAKRGHL
EIVEVLLKNGADVNAQDKFGKTAFDISIDNGNEDLAEILQKLGSGSGSMGVQVETISPGD
GRTFPKRGQTCVVHYTGMLEDGKKVDSSRDRNKPFKFMLGKQEVIRGWEEGVAQMSV
GQRAKLTISPDYAYGATGHPGIIPPHATLVFDVELLKLEASDYKDDDDKGDYKDDDDKG
DYKDDDDK

FKBP12^{F36V}-K27-3×FLAG tag

MGVQVETISPGDGRTFPKRGQTCVVHYTGMLEDGKKVDSSRDRNKPFKFMLGKQEVIR
GWEEGVAQMSVGQRAKLTISPDYAYGATGHPGIIPPHATLVFDVELLKLEGSGSGSGSDL
GKKLLEAARAGQDDEVRILMANGADVNAHDTFGFTPLHLAALYGHLEIVEVLLKNGAD
VNADDSYGRTPLHLAAMRGHLEIVEVLLKYGADVNAADEEGRTPLHLAAKRGHLEIVE
VLLKNGADVNAQDKFGKTAFDISIDNGNEDLAEILQKLASDYKDDDDKGDYKDDDDK
GDYKDDDDK

NbALFA-FKBP12^{F36V}-3×FLAG

MEVQLQESGGGLVQPGGSLRLSCTASGVTISALNAMAMGWYRQAPGERRVMVAAVSER
GNAMYRESVQGRFTVTRDFTNKMVSLQMDNLKPEDTAVYYCHVLEDRVDSFHDYWG
QGTQVTVSSGSGSGSMGVQVETISPGDGRTFPKRGQTCVVHYTGMLEDGKKVDSSRDR
NKPFKFMLGKQEVIRGWEEGVAQMSVGQRAKLTISPDYAYGATGHPGIIPPHATLVFDVE
LLKLEASDYKDDDDKGDYKDDDDKGDYKDDDDK

bioRxiv preprint doi: https://doi.org/10.64898/2026.06.08.730974; this version posted June 10, 2026. The copyright holder for this preprint (which was not certified by peer review) is the author/funder, who has granted bioRxiv a license to display the preprint in perpetuity. It is made available under a CC-BY 4.0 International license.

Nb139-FKBP12^{F36V}-3×FLAG

AQVQLQESGGGLVQAGGSLRLSCAASERTFSTYAMGWFRQAPGREREFLAQINWSGTT
TYYAESVKDRTTISRDNAKNTVYLEMNNLNADDTGIYFCAAHPQRGWGSTLGWTYWG
QGTQVTVSSGSGSGSMGVQVETISPGDGRTFPKRGQTCVVHYTGMLEDGKKVDSSRDR
NKPFKFMLGKQEVIRGWEEGVAQMSVGQRAKLTISPDYAYGATGHPGIIPPHATLVFDVE
LLKLEASDYKDDDDKGDYKDDDDKGDYKDDDDK

C10-FKBP12^{F36V}-3×FLAG

MGSDLGKKLLEAAWHGQDDEVRILMANGADVNATDQSGMTPLHLAAWRGHLEIVEVL
LKTGADVNAIDRWGKTPLHLAARIGHLEIVEVLLKAGADVNAQDKFGKTPFDLAIDNG
NEDIAEVLQKAAGSGSGSMGVQVETISPGDGRTFPKRGQTCVVHYTGMLEDGKKVDSS
RDRNKPFKFMLGKQEVIRGWEEGVAQMSVGQRAKLTISPDYAYGATGHPGIIPPHATLVF
DVELLKLEASDYKDDDDKGDYKDDDDKGDYKDDDDK

bioRxiv preprint doi: https://doi.org/10.64898/2026.06.08.730974; this version posted June 10, 2026. The copyright holder for this preprint (which was not certified by peer review) is the author/funder, who has granted bioRxiv a license to display the preprint in perpetuity. It is made available under a CC-BY 4.0 International license.

3. Supplementary figures

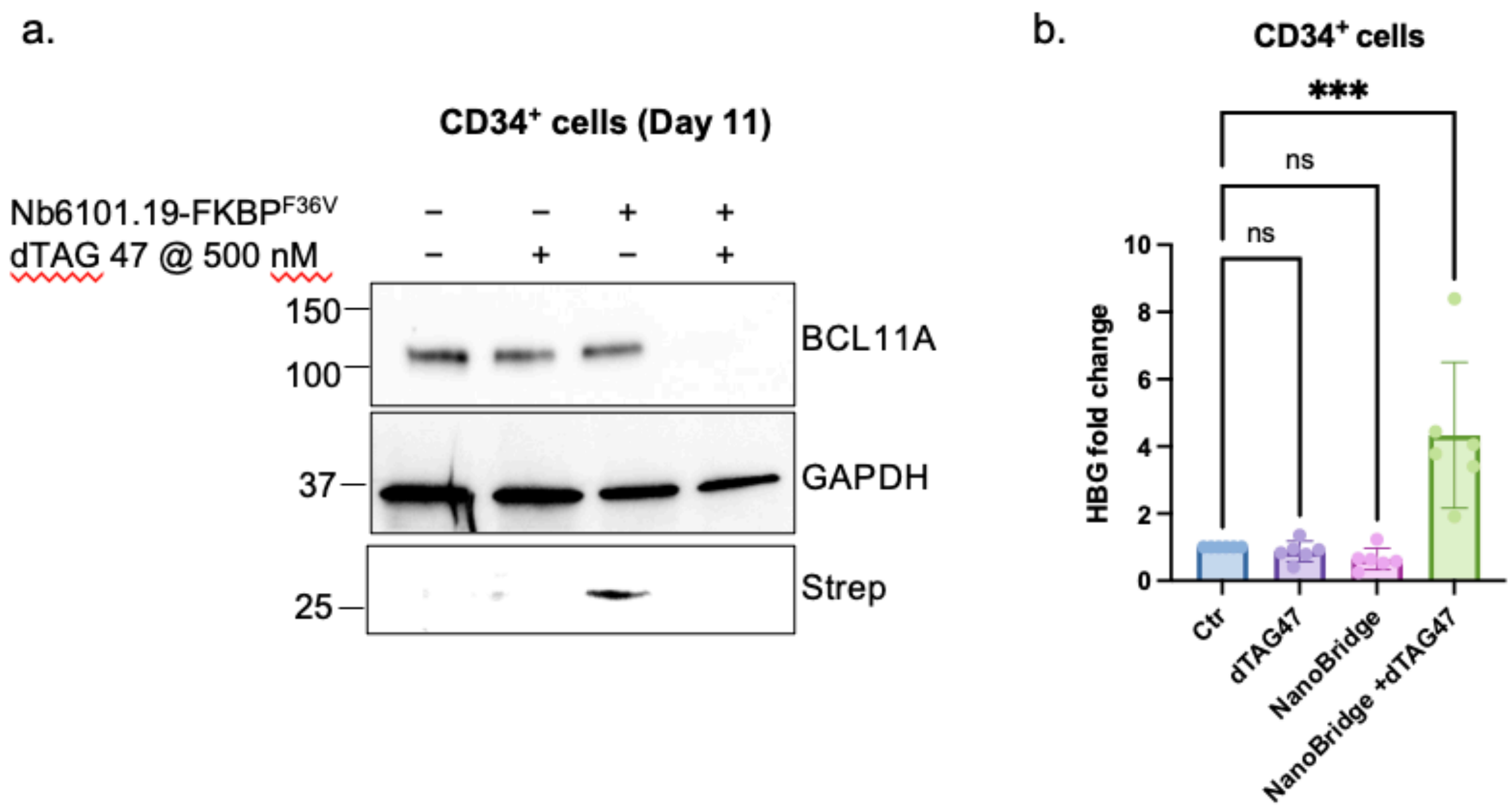

Figure S1. BCL11A degradation via the NanoBridge. (a) Immunoblots revealing that BCL11A degraded in $CD34^+$ cells only in the presence of both the NanoBridge (Nb6101.19-$FKBP^{F36V}$) and dTAG-47. (b) Fold change of HBG transcripts in $CD34^+$ cells treated as indicated. $CD34^+$ cells were collected from Day 13 and analyzed by RT-qPCR (mean ± s.d., n = 3, ***$P < 0.001$).

bioRxiv preprint doi: https://doi.org/10.64898/2026.06.08.730974; this version posted June 10, 2026. The copyright holder for this preprint (which was not certified by peer review) is the author/funder, who has granted bioRxiv a license to display the preprint in perpetuity. It is made available under a CC-BY 4.0 International license.

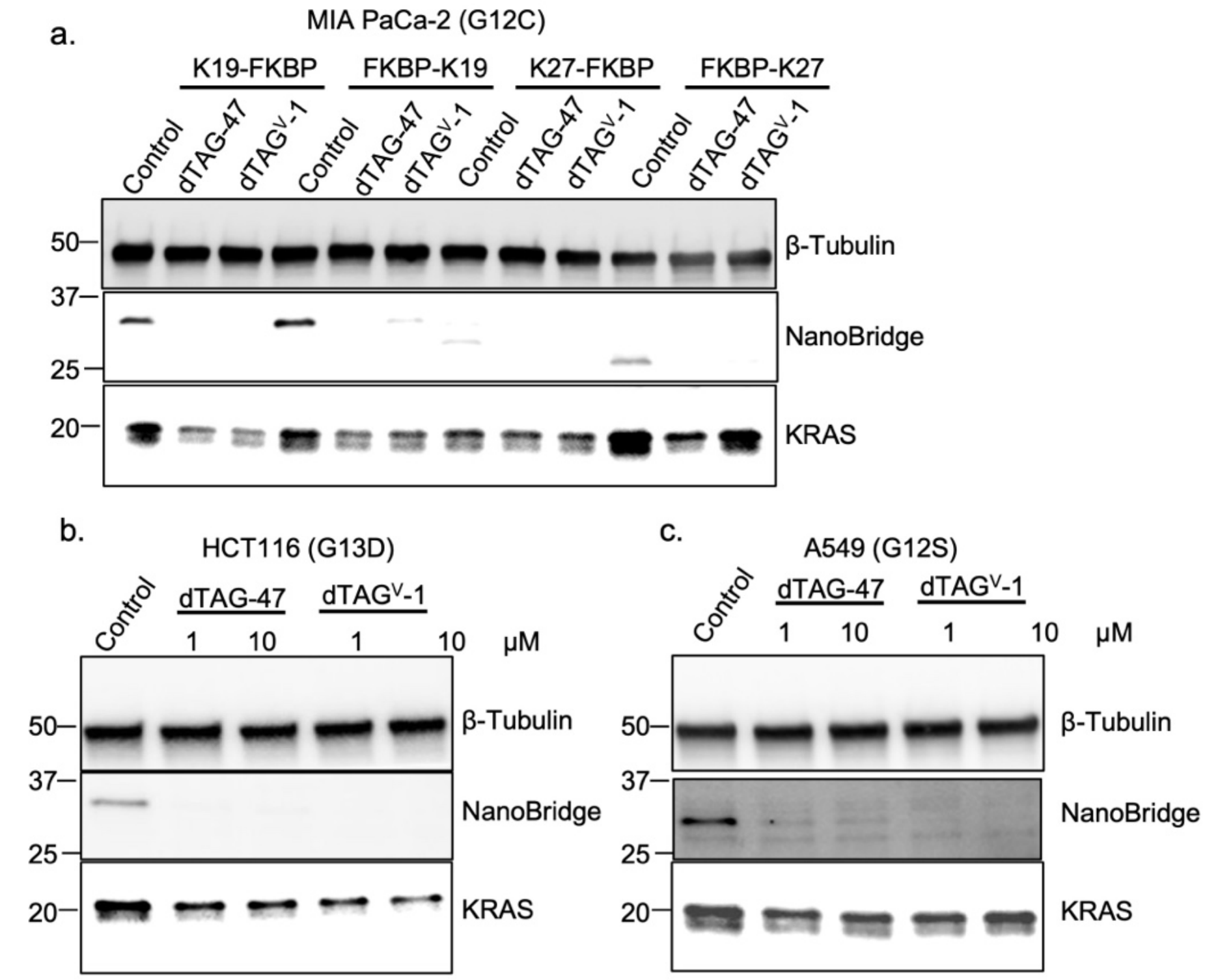

Figure S2. NanoBridge-induced targeted degradation of different KRAS mutants in cancer cells. Immunoblots revealing (a) degradation of KRAS (G12C) in Mia-PaCa 2 expressing different NanoBridge constructs upon treatment with 1 μM dATG47 or dTAG^{V}-1 for 24h, (b) degradation of KRAS (G13D) in HCT116 cells and (c) degradation of KRAS(G12S) in A549 cells upon treatment with 1μM or 10 μM dATG47 or dTAG^{V}-1 for 24h.

bioRxiv preprint doi: https://doi.org/10.64898/2026.06.08.730974; this version posted June 10, 2026. The copyright holder for this preprint (which was not certified by peer review) is the author/funder, who has granted bioRxiv a license to display the preprint in perpetuity. It is made available under a CC-BY 4.0 International license.

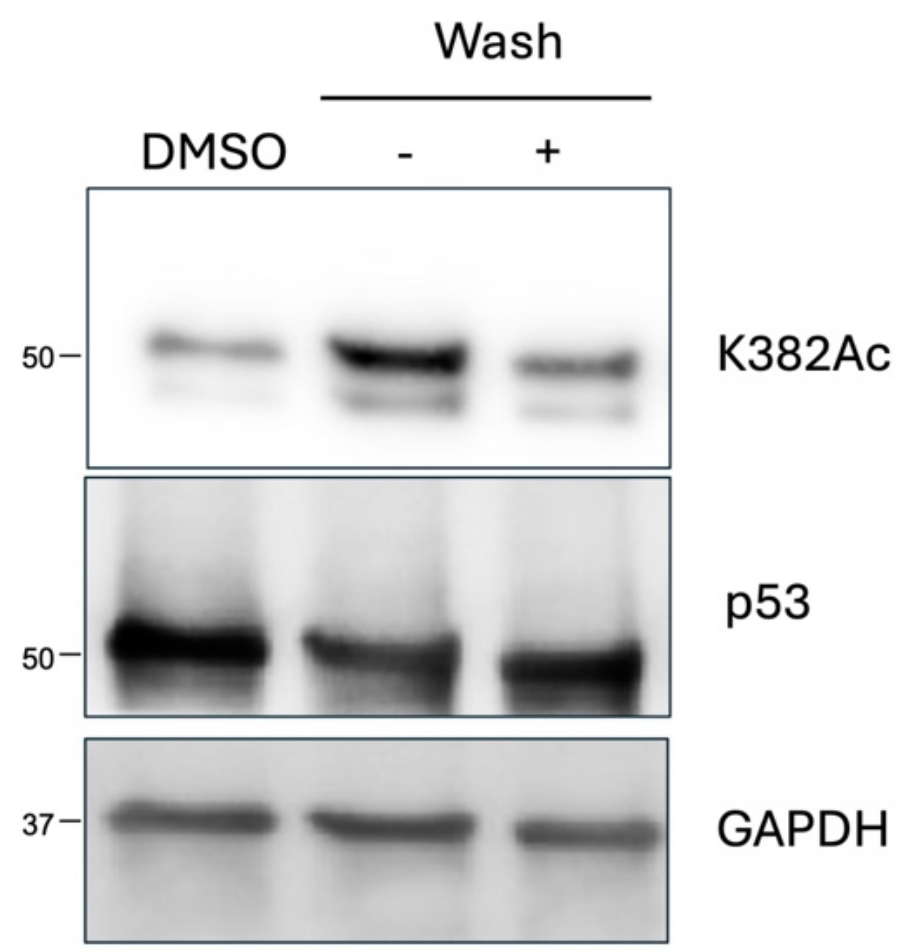

Figure S3. Reversibility of PTM induction. Immunoblots revealing removal of AceTAG-1 via a washout resulted in the reduction of p53 acetylation at K382 in 12 h. H1299 cells expressing Nb139-FKBP12^{K36V} were treated with 500 nM AceTAG-1 for 3 h, and cells were washed with PBS for 3 times, and cultured in fresh complete medium for another 12 h. The cells not washed were treated with 500 nM AceTAG-1 for 15 h.

bioRxiv preprint doi: https://doi.org/10.64898/2026.06.08.730974; this version posted June 10, 2026. The copyright holder for this preprint (which was not certified by peer review) is the author/funder, who has granted bioRxiv a license to display the preprint in perpetuity. It is made available under a CC-BY 4.0 International license.

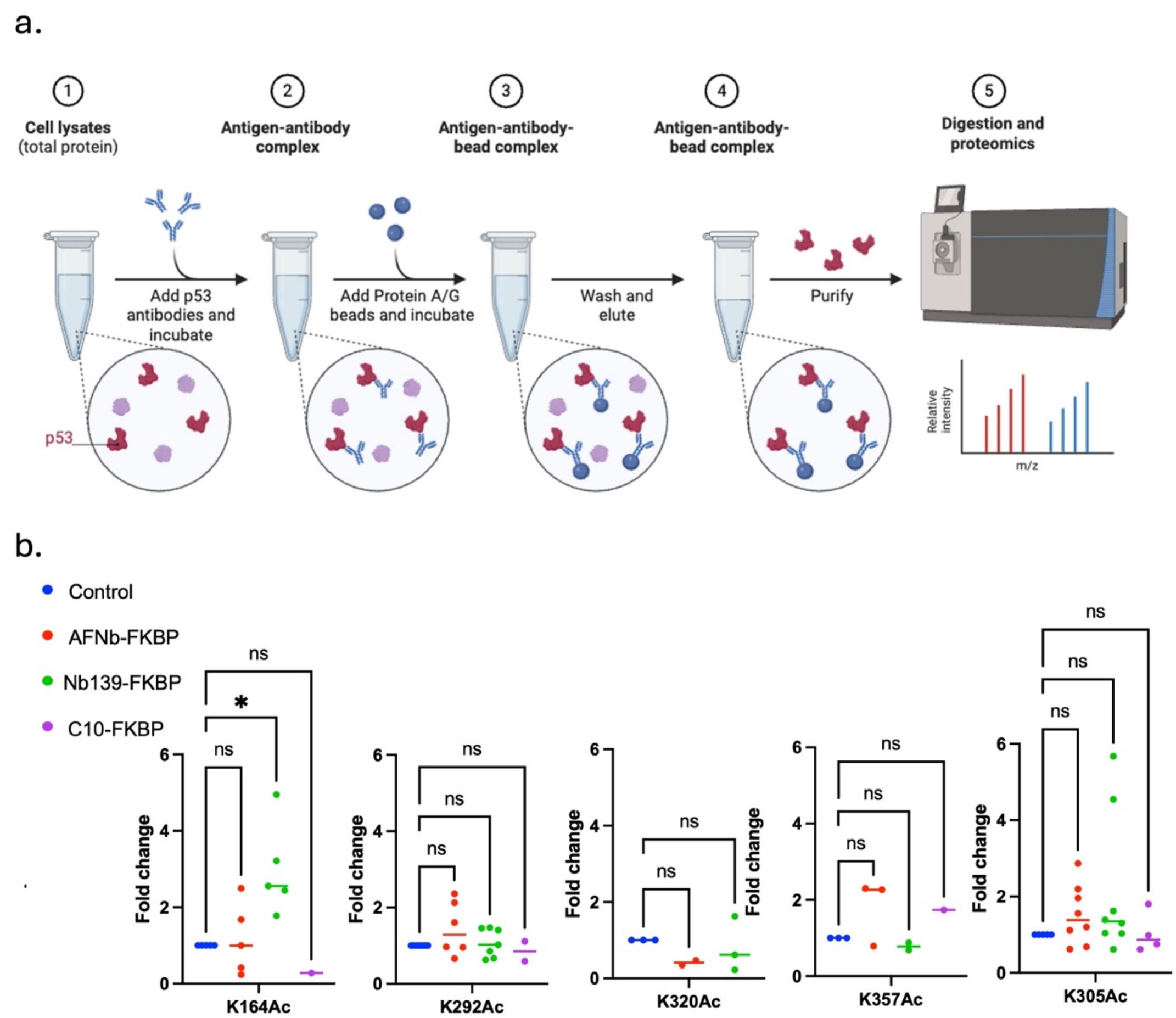

Figure S4. Proteomics to determine the fold change of p53 acetylated lysines in H1299 cells expressing different NanoBridge constructs treated with AceTAG-1. (a) Schematic description of the experimental workflow. Cells were treated with DMSO or AceTAG-1 (500 nM) for 16h and lysed; p53 was immunoprecipitated, digested and subjected to LC/MS/MS for analysis. (b) Fold change determined via mass spectrometry. Data represent a median of $n = 4$ biological replicates for AceTAG-1-treated cells. Note that peptides may not have been detected in every replicate but were detected and quantified in a minimum of two biological replicates to be included in this analysis. No statistically significant acetylation was detected for K292, K320, K357, and K305. Statistical significance was assessed via one-way ANOVA comparing DMSO-treated sample to AceTAG-1 treated samples. $*p < 0.05$; $***p < 0.001$; $****p < 0.0001$.

bioRxiv preprint doi: https://doi.org/10.64898/2026.06.08.730974; this version posted June 10, 2026. The copyright holder for this preprint (which was not certified by peer review) is the author/funder, who has granted bioRxiv a license to display the preprint in perpetuity. It is made available under a CC-BY 4.0 International license.

4. Supplementary tables

Table S1. Primers used for plasmid construction

Plasmid	Primer	Sequence (5'-3')
Nb6101.16-FKBP12^{F36V}-NLS-Strep	Vector- FWD	CGGCAGCTGGAGCCA
	Vector- REV	CATGGTGAATTCACCGGAAATAGATCC
2D9-FKBP12^{F36V}-NLS-Strep	2D9-FWD	GAATTCACCATGCAAGTTCAGCTGGTTG
	2D9-REV	AACCGCTGCCGCTGGAAACGGTAAC
	Vector-FWD	TCCAGCGGCAGCGGTTCAGGCT
	Vector-REV	CATGGTGAATTCACCGGAAATAGATCC
45S-FKBP12^{F36V}-NLS-Strep	Nb45S-FWD	TCCGGTGAATTCACCATGCGAGTTCAGCTTGTA
	Nb45S-REV	AGCCTGAACCGCTGCCCGATGATACAGTTACTTGGGT
	Vector-FWD	GGCAGCGGTTCAGGC
	Vector-REV	CATGGTGAATTCACCGGAAA
K19-FKBP12^{F36V}-3×FLAG	Insert-FWD	GACCTTGGAAAGAAACTTTTGGAAG
	Insert-REV	TTGAGCCTGAACCGCTGCCCAACTTCTGGAGTATTTCGGCGAGG
	Vector-FWD	GGCAGCGGTTCAGGCTCAATGG
	Vector-REV	CTTCCAAAAGTTTCTTTCCAAGGTCCATGGTGAATTCACCGGAAATAGATCCTCACG
FKBP12^{F36V}-K19-3×FLAG	Vector-FWD	GGCCCGTTTAAGGATCCCGC
	Vector-REV	GGTGAATTCACCGGAAATAGATCC
K27-FKBP12^{F36V}-3×FLAG	Vector-FWD	GGCCCGTTTAAGGATCCCGC
	Vector-REV	GGTGAATTCACCGGAAATAGATCC
FKBP12^{F36V}-K27-3×FLAG	Insert-FWD	TGGTAGTGGCTCCGGAAGCGATTTGGGCAAAAAGCTCCTTGAG
	Insert-REV	ATCGTCATCGTCTTTGTAATCGCTAGCGAGCTTTTGCAGAATTTCTGCGAG
	Vector-FWD	GCTAGCGATTACAAAGACGATGACGATAAGGA
	Vector-REV	GCTTCCGGAGCCACTACCACTGCC
	Vector-FWD	GGCAGCGGTTCAGGCTCAATGG

bioRxiv preprint doi: https://doi.org/10.64898/2026.06.08.730974; this version posted June 10, 2026. The copyright holder for this preprint (which was not certified by peer review) is the author/funder, who has granted bioRxiv a license to display the preprint in perpetuity. It is made available under a CC-BY 4.0 International license.

NbALFA-FKBP12^{F36V}-3×FLAG	Vector-REV	GGTGAATTCACCGGAAATAGATCCTCACG
Nb139-FKBP12^{F36V}-3×FLAG	Insert1-FWD	ATCTATTTCCGGTGAATTCACCATGGAGGAGCCGCAGTCAGATCCT
	Insert1-REV	CTCGAGGGGTCCGGGATTCTC
	Insert2-FWD	GAGAATCCCGGACCCCTCGAGGCACAGGTGCAACTCCAAGA
	Insert2-REV	GCGGGATCCTTAAACGGGC
	Vector-FWD	GGCCCGTTTAAGGATCCCGC
	Vector-REV	GGTGAATTCACCGGAAATAGATCC
C10-FKBP12^{F36V}-3×FLAG	Vector-FWD	GGCAGCGGTTCAGGCTCAATGG
	Vector-REV	GGTGAATTCACCGGAAATAGATCCTCACG

**Table S2. Primes used for RT-qPCR**	
HBB-FWD:	CTGAGGAGAAGTCTGCCGTTA
HBB- REV	AGCATCAGGAGTGGACAGAT
HBE- FWD	GCAAGAAGGTGCTGACTTCC
HBE- REV	ACCATCACGTTACCCAGGAG
HBG- FWD	TGGATGATCTCAAGGGCAC
HBG- REV	TCAGTGGTATCTGGAGGACA
GAPDH- FWD	ACCCAGAAGACTGTGGATGG
GAPDH-REV	TTCAGCTCAGGGATGACCTT

bioRxiv preprint doi: https://doi.org/10.64898/2026.06.08.730974; this version posted June 10, 2026. The copyright holder for this preprint (which was not certified by peer review) is the author/funder, who has granted bioRxiv a license to display the preprint in perpetuity. It is made available under a CC-BY 4.0 International license.

Table S3. C_q values of hemoglobin transcripts in CD34$^+$ cells.

**C_q values of hemoglobin transcripts in CD34$^+$ cells expressing Nb6101.19-FKBP12^{F36V}**												
**CD34$^+$ cells (Day 13)**												
	Untreated						Nb6101.19-FKBP12^{F36V}					
	Cq 1	Cq 2	Cq 3	Cq Avg	DCq	2^{-DCq}	Cq 1	Cq 2	Cq 3	Cq Avg	DCq	2^{-DCq}
HBB	23.09	23.03	22.95	23.02	-0.43	1.35	26.08	25.69	26.03	25.93	1.88	0.27
HBG	23.31	23.06	23.28	23.21	-0.23	1.17	24.07	24.06	24.47	24.2	0.15	0.90
GAP DH	23.76	23.3	23.28	23.45	/	/	24.27	23.88	24.01	24.05	/	/
**C_q values of hemoglobin transcripts in CD34$^+$ cells expressing different NanoBridge constructs**												
**CD34$^+$ cells (Day 13)**												
	Control						dATG47					
	Cq 1	Cq 2	Cq 3	Cq Avg	DCq	2^{-DCq}	Cq 1	Cq 2	Cq3	Cq Avg	DCq	2^{-DCq}
HBB	17.58	17.50	17.84	17.64	-6.93	122.22	17.24	17.08	17.08	17.13	-7.40	169.29
HBG	20.64	20.85	20.88	20.79	-3.78	13.77	20.63	20.85	20.88	20.79	-3.75	13.45
GAP DH	24.50	24.37	24.85	24.57	/	/	24.39	24.44	24.78	24.54	/	/
	NbGFP-FKBP12^{F36V}						Nb6101.19-FKBP12^{F36V}					
	Cq 1	Cq 2	Cq 3	Cq Avg	DCq	2^{-DCq}	Cq 1	Cq 2	Cq3	Cq Avg	DCq	2^{-DCq}
HBB	19.16	18.97	19.00	19.04	-6.37	82.71	19.61	19.33	19.86	19.6	-6.10	68.44
HBG	22.38	22.36	22.58	22.44	-2.97	7.85	21.68	21.78	22.04	21.83	-3.86	14.55
GAP DH	25.17	25.02	26.05	25.41	/	/	25.72	25.66	25.71	25.70	/	/
	Nb2D9-FKBP12^{F36V}						Nb45S-FKBP12^{F36V}					
	Cq 1	Cq 2	Cq 3	Cq Avg	DCq	2^{-DCq}	Cq 1	Cq 2	Cq3	Cq Avg	DCq	2^{-DCq}
HBB	19.12	18.51	18.81	18.81	-6.79	110.40	19.46	19.27	19.37	19.37	-5.95	61.82
HBG	21.34	21.17	21.40	21.30	-4.30	19.65	21.31	21.51	21.69	21.50	-3.81	14.06
GAP DH	25.50	25.46	25.84	25.6	/	/	25.29	25.19	25.47	25.32	/	/

bioRxiv preprint doi: https://doi.org/10.64898/2026.06.08.730974; this version posted June 10, 2026. The copyright holder for this preprint (which was not certified by peer review) is the author/funder, who has granted bioRxiv a license to display the preprint in perpetuity. It is made available under a CC-BY 4.0 International license.

5. References.

(1) Kurita, R.; Suda, N.; Sudo, K.; Miharada, K.; Hiroyama, T.; Miyoshi, H.; Tani, K.; Nakamura, Y. Establishment of Immortalized Human Erythroid Progenitor Cell Lines Able to Produce Enucleated Red Blood Cells. *PLOS ONE* **2013**, *8* (3), e59890. https://doi.org/10.1371/journal.pone.0059890.
(2) Giani, F. C.; Fiorini, C.; Wakabayashi, A.; Ludwig, L. S.; Salem, R. M.; Jobaliya, C. D.; Regan, S. N.; Ulirsch, J. C.; Liang, G.; Steinberg-Shemer, O.; Guo, M. H.; Esko, T.; Tong, W.; Brugnara, C.; Hirschhorn, J. N.; Weiss, M. J.; Zon, L. I.; Chou, S. T.; French, D. L.; Musunuru, K.; Sankaran, V. G. Targeted Application of Human Genetic Variation Can Improve Red Blood Cell Production from Stem Cells. *Cell Stem Cell* **2016**, *18* (1), 73–78. https://doi.org/10.1016/j.stem.2015.09.015.
(3) Benskey, M. J.; Manfredsson, F. P. Lentivirus Production and Purification. In *Gene Therapy for Neurological Disorders: Methods and Protocols*; Manfredsson, F. P., Ed.; Springer: New York, NY, 2016; pp 107–114. https://doi.org/10.1007/978-1-4939-3271-9_8.
